# Pan-Cancer Multiomics Analysis of *TC2N* Gene Suggests its Important Role(s) in Tumourigenesis of Many Cancers

**DOI:** 10.31557/APJCP.2020.21.11.3199

**Published:** 2020-11

**Authors:** Muhammad Asif Qureshi, Saeed Khan, Muhammad Sohaib Tauheed, Sofia Ali Syed, Ikram Din Ujjan, Amanullah Lail, Shaheen Sharafat

**Affiliations:** 1 *Department of Pathology, Dow International Medical College, Dow University of Health Sciences Karachi, Pakistan. *; 2 *Department of Oral Pathology, Dow Dental College, Dow University of Health Sciences, Karachi, Pakistan. *; 3 *Department of Pathology, Liaquat University of Medical and Health Sciences Jamshoro, Pakistan. *; 4 *Department of Paediatrics, Dow University of Health Sciences Karachi, Pakistan. *

**Keywords:** TC2N, Tac2-N, TCGA, GTEx, cancer

## Abstract

**Background:**

Role of *TC2N* in carcinogenesis has been largely unfathomed until recently when it was identified as a novel oncogene in lung cancer. Subsequently, a tumour suppressor role of *TC2N* was reported in breast cancer. It is therefore highly relevant to investigate *TC2N* molecular partners/mechanisms on a larger scale including a wider range of tumour types.

**Methods:**

We investigated *TC2N* mRNA expression, its promoter methylation levels, effects of *TC2N* transcription on overall patient survival, somatic mutations in *TC2N* gene and correlation between *TC2N *mRNA expression and other cancer genes in pan-cancer by using data available from the Cancer Genome Atlas (TCGA) and the Genotype Tissue Expression (*GTEx*) databases.

**Results:**

*TC2N* mRNA expression was differentially regulated in 9/33 TCGA tumour types. Of these 9 tumours, 5 tumour types (cholangiocarcinoma, ovarian-serous-cystadenocarcinoma, rectal-adenocarcinoma, stomach-adenocarcinoma and thymoma) had significantly higher *TC2N *mRNA expression while 4 (pheochromocytoma-and-paraganglioma, skin-cutaneous-melanoma, thyroid-carcinoma and uterine-carcinosarcoma) had significantly lower *TC2N* mRNA expression compared to matched and normal controls. *TC2N *promoter was hypermethylated in most cancers while hypomethylated in head-and-neck-squamous-cell-carcinoma and kidney-renal-clear-cell carcinoma. *TC2N* transcription was positively correlated with transcription of several other cancer genes including genes from *Myc, cell-cycle, Nrf2, Wnt, PI3K, Hippo, Notch, TGFβ* and *RAS/RTK* pathways. Poor prognosis was associated with higher *TC2N* mRNA levels in pancreatic-adenocarcinoma and brain-lower-grade-glioma and lower *TC2N *mRNA levels in kidney-renal-clear-cell-carcinoma, mesothelioma, sarcoma and skin-cutaneous melanoma. Functional protein partners of *TC2N* were identified as *STX2, SMEK1, SMEK2, STXBP5, SCARA5, MMRN1, CATSPER2, CATSPERB, CLEC4M *and *STAB2*. Many of these proteins are key players in carcinogenesis of various cancers. Highest pathogenic somatic mutation rates in *TC2N* were found in skin-cutaneous-melanoma, uterine-corpus-endometrial-carcinoma, colon-endocervical-adenocarcinoma, bladder-urothelial-carcinoma and breast-invasive-carcinoma.

**Conclusion:**

Our findings unravel several un-explored avenues related to the role of *TC2N* in tumourigenesis of several cancers, suggesting *TC2N* as an important player and a potential candidate for tumour-therapy.

## Introduction

*Tac2-N (TC2N)* gene is located on chromosome 14a32.12 and encodes a C2 domain containing protein that belongs to the carboxyl terminal type (C-type) tandem C2 family of proteins. The protein contains two C-terminal C2 domains, C2A and C2B (Fukada and Mikoshiba, 2001). Initially, the C2 domains were identified as protein structural domains for calcium-dependent protein kinase C (Duncan et al., 2000; Corbalan-Garcia et al., 2014). However, further data delineated other roles of C2 domains including cellular signal transduction and other protein-protein interactions (Farah and Sossin, 2012). Interestingly, various proteins containing the C2 domain have been linked to regulation of carcinogenesis. For example, DOC2B is believed to play a tumour suppressive role in cervical cancer by inhibition of cellular proliferation, migration and invasion of malignant cells (Kabekkodu et al., 2014). Another C2 domain containing protein, Myoferlin, promotes tumour metastasis in patients with triple negative breast cancer (Blommme et al., 2017). 

Role of TC2N in tumourigenesis had not been investigated until recently when it was identified as a novel oncogene that promotes/accelerates tumourigenesis by suppression of p53 signaling in lung cancer (Hao et al., 2019). Subsequently, the same group reported TC2N as a potent suppressor of PI3K-AKT signaling in breast cancer, suggesting its tumour suppressor activity in breast cancer (Hao et al., 2019). While these recent (and only) studies have highlighted TC2N as a potential player in lung and breast cancers, its role in other tumour types remains un-addressed. It is therefore highly relevant to investigate TC2N molecular profile and associated circuitry to better understand its role in various cancers.

In this study, we undertook multiomics approach to investigate TC2N molecular profile in pan-cancer using tumour data sets available at the The Cancer Genome Atlas (TCGA). In this study, we investigated *TC2N *mRNA expression, its promoter methylation status, effect of TC2N transcription on patients’ prognosis, correlation between *TC2N* mRNA expression and other cancer genes, TC2N functional protein partners and somatic mutations in *TC2N* gene in pan-cancer. This, to the best of our knowledge, is the first report detailing pan-cancer analysis of TC2N molecular profile. Our results provide novel insights with reference to TC2N molecular interactions and its potential role in carcinogenic mechanisms. These findings could therefore be exploited not only to better understand underlying cancer mechanisms but also to identify novel biological targets for cancer treatment.  

## Materials and Methods

*Methods*

*TC2N transcript expression analyses using GEPIA and UALCAN*

*TC2N* transcript expression analysis in 33 tumour types available in the *TCGA* database was carried out using the Gene Expression Profiling Interactive Analysis (GEPIA), which is an open-source web platform to analyze RNA sequencing data (http://gepia.cancer-pku.cn/) (Tang et al., 2017). The GEPIA analyses are based on data extraction from the TCGA (tumour data sets) and GTEx (normal controls) databases providing an additional benefit of including normal tissues in the expression analyses (from the *GTEx* database). Bar graphs for *TC2N* gene expression were plotted for pan-cancer (33 tumour types) and matched as well as normal controls from the GTEx database. Subsequently, box plots were generated for tumours with differential *TC2N* expression using a Log2 (TPM+1) scale, p-value cut-off of 0.01, a LogFc cutoff of 1. For the 9 tumour types exhibiting differential expression patterns at the *GEPIA* platform, further in-depth transcript expression analyses (with reference to tumour subtypes, different stages, different grade, nodal metastasis and other available clinical details) were performed using the UALCAN (http://ualcan.path.uab.edu/), which is an open-access web server that provides detailed analyses of TCGA datasets (including normal control tissues from the GTEx database) (Chandrashekar et al., 2017). It is important to note that all clinical/sample details were not available for all tumour types and therefore analyses of available parameters were performed. Therefore, of the 9 tumour types that showed differential TC2N transcript expression, 5 (*READ, OV, THYM, SKC* and *UCS*) were excluded from the UALCAN analyses because of one (or more) of the following reasons; n<3 in any of the sample type, non-availability of the GTEx data for comparison, or unavailability of relevant parameter for comparison. For these reasons, tumour stage-specific anlyses could only be performed for CHOL, STAD and THCA while analyses with other clinicopathological parameters was performed for CHOL, STAD, THCA, OV and PCPG. 

*TC2N promoter methylation in pan-cancer*

TC2N promoter methylation levels were investigated in pan-cancer using an open-source web-based human pan-cancer methylation database (MethHC) [http://methhc.mbc.nctu.edu.tw/php/index.php] (Huang et al., 2015). MethHC provides an interactive platform for analyses of DNA methylation, microRNA methylation, and correlation of expression and methylation data across 18/33* TCGA *tumour types. At the time of analyses, DNA methylation profiles available at the MethHC were based on integration of data from over 6000 samples, 6,548 microarray and >12,000 RNA-seq data from 18/33 *TCGA* tumour types. Box plots for *TC2N* promoter methylation were generated *TC2N* transcript (Accession #: NM_001128596) data from the TCGA database.

*Investigating prognostic significance of TC2N transcription on patients’ survival*

Based on *TC2N* expression, overall patient survival analyses were performed using Log Rank test (Mantel-Cox test). In order to stratify tumour cohorts into patients with high and low *TC2N* expression, *TC2N* expression threshold of 50% (median value) was used as a cut-off. All 33 cancers were investigated for effects of *TC2N *transcription patterns on patients’ survival/prognosis. Only those curves are presented herein which showed significant differences in overall survival (p<0.05) of patients with high cut-off values compared to those with low cut-off values.

*Correlation analyses of TC2N transcript with other cancer genes*

Pair-wise transcript expression analyses were performed on *TCGA* and *GTEx* data sets using Pearson’s correlation coefficient. Initially, we investigated very strong, strong, moderate and week correlations of *TC2N *expression with other genes in all 33 cancers at the *TCGA* database. Genes exhibiting very strong *TC2N* correlation (R>0.8) and strong negative correlations are shown herein. We also investigated correlation of *TC2N* expression with various tumour suppression genes including *BUB1B, CYLD, ATR, ATM, BRCA1, BRCA2, TP53*. Next, we investigated TC2N transcript correlation with various genes of 10 cancer signaling pathways, i.e, Myc pathway, cell cycle pathway, p53 pathway, Nrf2 pathway, Wnt pathway, Hippo pathway, TGFβ pathway, Notch pathway, PI3K pathway, RTK/RAS pathway. Following brackets (based on R-value) were used to interpret the +ve correlation data: R-value”; R>0.8 as very strong correlation; R=0.6-0.79 as strong correlation, R=0.4-0.59 as moderate correlation, R=0.2-0.39 as week correlation and R<0.2 as very week correlation.

*Protein-Protein-Interaction analyses using STRING*

In order to identify functional protein partners of TC2N, the Search Tool for the Retrieval of Interacting Genes/proteins (STRING) was used, which is an open-access web based tool that provides critical analyses of protein-protein interaction including direct (physical) as well as indirect (function) protein associations (https://string-db.org/) (Szklarcyzk et al., 2019). At the time of analyses presented herein, the STRING database contained data from a total of 5,090 organisms, 24,584,628 proteins and 3,123,056,667 protein-protein interactions. The TC2N protein network was constructed using neighborhood, gene fusion, co-expression, experiments, text-mining approaches.

*TC2N mutation profiling using the GDC Data Portal*

Mutation profiling of *TC2N* gene was performed using the National Cancer Institute’s GDC Data Portal, which is a partially open-access platform for online analyses and interactive visualization of cancer data-sets (https://portal.gdc.cancer.gov/) (Liu et al., 2018). It contains various types of mutation data including copy number variants, types of mutation and limited anonymized clinical data. At the time of investigation, data from 52 cancer projects, 34,893 cases, 22,872 genes and 3,142,246 mutations were available at the GDC data portal for analyses. For mutation, it provides mutation ID, details of genetic change, protein change, type of mutation and its VEP impact across all available TCGA tumour data sets. It also provides external links to dbSNP and Catalogue of Somatic Mutations in Cancer (COSMIC) database. Clinical impact of the SNVs was identified using Functional Analysis through Hidden Markov Models (FATHMM) with a score of >0.8 annotated as “pathogenic” (Shihab et al., 2015). 

## Results

*TC2N mRNA expression is differentially regulated in 9/33 TCGA tumour types*

**Figure 1:**
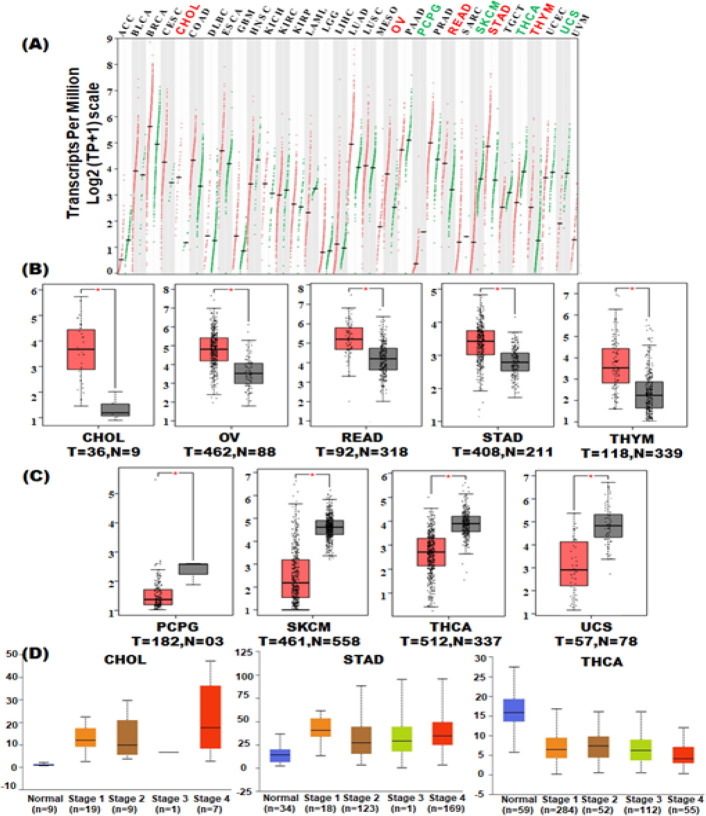
*TC2N *Gene Expression in Pan-Cancer. TCGA RNA-seq data were normalized (Transcripts per million, TPM), and differential expression was assessed using an unpaired t-test by comparing the tumour samples with normal controls (TCGA normal and GTEx databases respectively). (A) Expression of TC2N in pan-cancer. (B) Tumour types exhibiting significant overexpression of TC2N. (C) Tumour types exhibiting significant downregulation of TC2N expression. (D) Stage-specific expression analysis

In order to investigate *TC2N* mRNA expression patterns, data from TCGA database were analyzed across 33 cancer types compared to matched normal tissues from the TCGA database as well normal controls available at the GTEx database. TC2N transcription was significantly higher in cholangiocarcinoma (CHOL), ovarian serous cystadenocarcinoma (OV), rectal adenocarcinoma (READ), stomach adenocarcinoma (STAD) and thymoma (THYM) (Figure 1A, Figure 1B). TC2N transcription was significantly lower in pheochromocytoma and paraganglioma (PCPG), skin cutaneous melanoma (SKCM), thyroid carcinoma (THCA) and uterine carcinosarcoma (UCS) (Figure 1A, Figure 1C). Boxplots presented in Figure 1B and Figure 1C were generated only for those TCGA tumours which exhibited significantly differential expression as compared to controls. 

We further investigated TC2N expression patterns with respect to tumor stages, tumour grades, histological subtypes, nodal metastasis and other clinical parameters only in those 9 tumours which showed significant differential expression of *TC2N* (i.e, CHOL, OV, READ, STAD, THYM, PCPG, SKCM, THCA and UCS). Amongst hepatobilliary tumours, CHOL showed significantly increased *TC2N* expression in all tumour stages compared to the controls (Figure 1D, Table 1). Moreover, *TC2N* expression was also significantly higher in CHOL cases with nodal metastasis as compared to the controls (Table 1). Amongst the gastrointestinal tract tumours, STAD showed significantly increased *TC2N *expression in all stages, tumour grades and histological subtypes compared to the normal controls (Table 1). Moreover, *TC2N* expression in STAD was significantly higher in patients with nodal metastasis and those without H.pylori infection (Table 1). Amongst the tumours with low overall *TC2N* expression, THCA showed significant downregulation of TC2N in all histologic subtypes compared to controls while in PCPG only Paraganglioma;extra adrenal pheochromocytoma subtype showed significant downregulation of TC2N compared to the controls (Table 1). 

Taken together, these data exhibit first ever pan-cancer expression analyses for *TC2N *expression and therefore delineate possible role of* TC2N* in these cancers. 

*Variation of promoter methylation levels of TC2N in Pan-Cancer*

**Figure 2:**
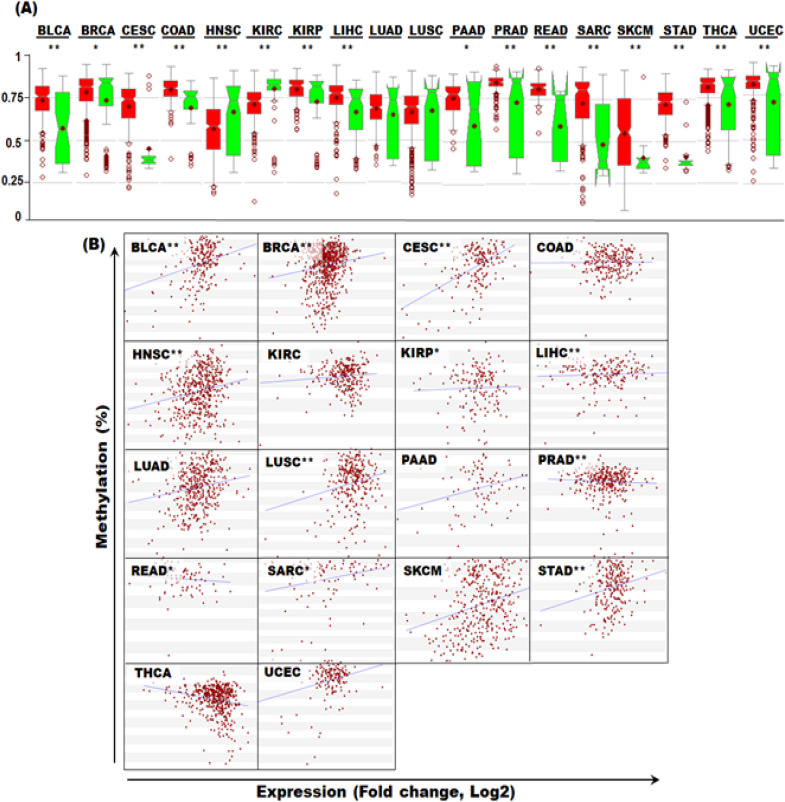
Promoter Methylation of *TC2N* Gene in Pan-Cancer. (A) Promoter methylation levels of *TC2N* gene in pan-cancer. (B) Correlation between *TC2N* mRNA expression and DNA methylation in Pan-Cancer

In order to investigate methylation of TC2N promoter in pan-cancer, MethHC database was used. Compared to the controls, TC2N promoter was significantly hypermethylated in BLCA, BRCA, COAD, KIRP, LIHC, PAAD, PRAD, READ, SARC, SKCM, STAD, THCA and UCEC and significantly hypomethylated in HNSC and KIRC (Figure 2A). We further investigated correlation between *TC2N* mRNA expression and its promoter methylation levels in pan-cancer. Significant +ve correlation was observed in *BLCA, BRCA, CESC, HNSC, KIRP, LIHC, LUSC, SARC* and *STAD* while significant –ve correlation was observed in *PRAD* and *READ* (Figure 2B).

*Role of TC2N mRNA levels in prognosis/patient survival*

**Figure 3:**
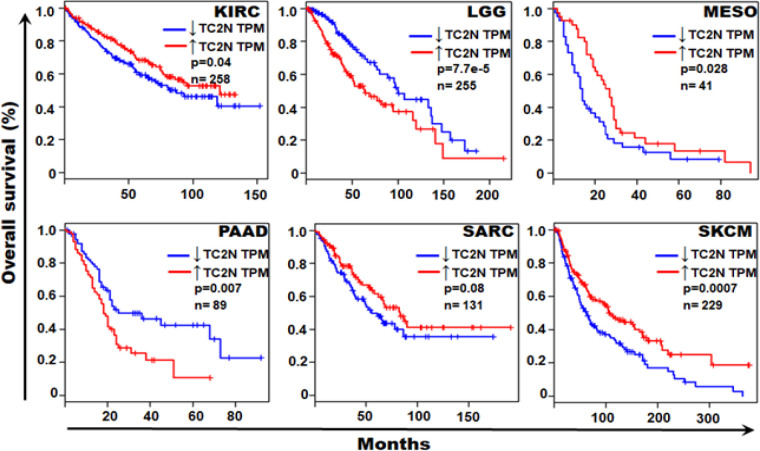
TC2N Expression as a Prognostic Factor in Various Tumours. Patients with survival data in the TCGA datasets were segregated into those with high TC2N and low TC2N expression levels (using TPM as the unit of gene expression). Overall survival was compared between TC2N high-expression levels and low-expression level patients. Red line represents tumours expressing high levels of TC2N transcripts while the blue lines represents tumours with low level TC2N transcript expression. Of the 33 TCGA tumours that were investigated for survival analyses, only 6 (shown in the figure) exhibited significant impact of TC2N expression levels on patients’ survival. TPM, transcript per million

In order to investigate potential role of TC2N mRNA levels in tumour prognosis and patient survival, Kaplan-Mayer curves were generated for pan-cancer using tumour data sets from the TCGA database. Of all the 33 tumour types, 6 tumours exhibited significant role of TC2N mRNA levels in tumour prognosis. Patients with higher TC2N mRNA levels showed a shorter overall survival (and thus poor prognosis) in LGG and PAAD tumour types (Figure 3). Patients with lower *TC2N* expression showed a shorter overall survival (and thus poor prognosis) in *KIRC, MESO, SARC* and *SKCM *tumour types (Figure 3). 

*TC2N mRNA expression is positively correlated with genes of various cancer pathways*

We investigated *TC2N* expression correlation with other cancer genes in all 33 cancers enlisted in the TCGA database. Of these 33 cancers, 32 tumours expressed genes showing +ve correlation and 28 tumours expressed genes exhibiting –ve correlation with *TC2N* expression. Of the 32 cancers showing +ve correlation with TC2N expression, 8 tumour showed very strong +ve correlation (Table 2). Of the 28 tumour expressing genes with –ve correlation with *TC2N* expression, 5 tumour types showed strong –ve correlation.

We further investigated *TC2N* expression correlation with several tumour suppressor genes including *BUB1* mitotic checkpoint serine-threonine kinase B (BUB1B), checkpoint kinase 2 (CHK2), *BRCA1, BRCA2ATM *serine-threonine kinase (ATM), ATR, tumour protein p53 (*TP53*), *CYLD* and *TSPYL2. BUB1B* showed +ve correlation with *TC2N* gene expression in *PCPG, HNSC, READ* and *PRAD. CYLD* showed +ve correlation with the following cancers: *DLBC, KICH, PRAD, UCEC, READ, TGCT, UVM, LAML, THCA, LIHC, THYM, KIRC, KIRP* and *SKCM*. With reference to *BRCA 1* and *BRCA1*, the earlier showed +ve correlation with only CECS while the later showed +ve correlation in the following cancers: CESC, HNSC, READ and DLBC. None of the investigated tumour suppressor genes showed -ve correlation with *TC2N* expression other than TP53 which showed –ve correlation only in UVM (Table 2).

**Figure 4:**
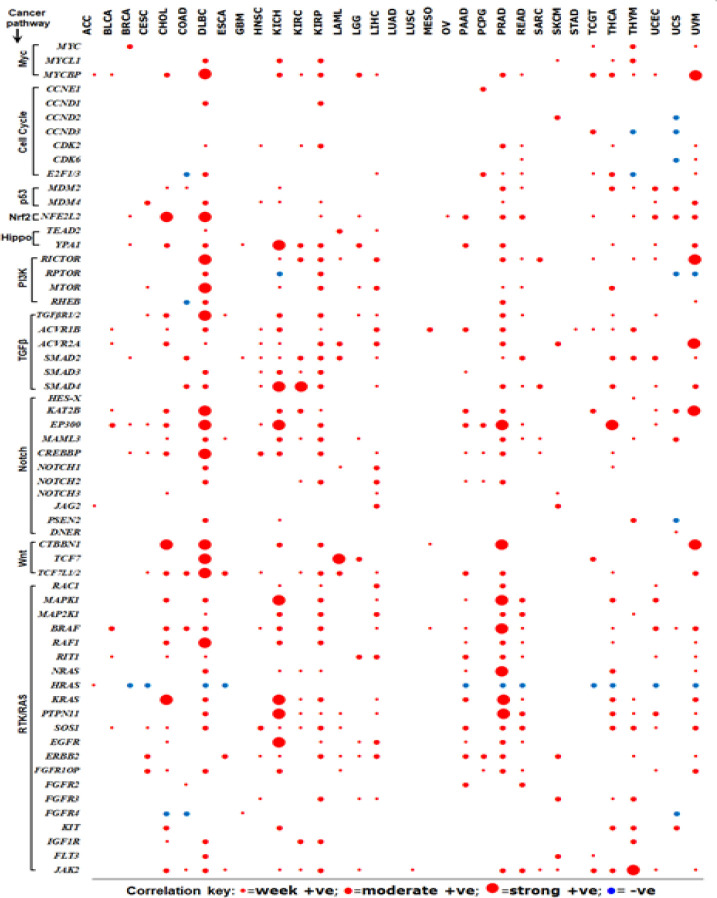
Correlation between TC2N Expression and Other Genes in Various Cancer Pathways. Correlation between TC2N mRNA expression and other genes in 10 common cancer pathways was investigated using Pearson’s correlation coefficient. Positive correlation brackets were identified as week (R=0.2-0.39), moderate (R=0.4-0.6) and strong (R>0.6) correlation. In this figure, strong and very strong positive correlation were drawn cumulatively and represented as strong positive correlation

In order to further understand the molecular circuitry involved in TC2N molecular loops, we investigated correlation between *TC2N* expression and selected genes in 10 cancer pathways namely, Myc pathway, cell cycle pathway, *p53* pathway, *Nrf2 *pathway, Wnt pathway, Hippo pathway, *TGFβ *pathway, Notch pathway, *PI3K *pathway and *RTK/RAS* pathway. It is interesting to note that several genes involved in the aforementioned cancer pathways show +ve correlation with *TC2N* expression (Figure 4), suggesting a potential role of *TC2N* in carcinogenesis of many cancers. 

*TC2N protein network*

**Figure 5:**
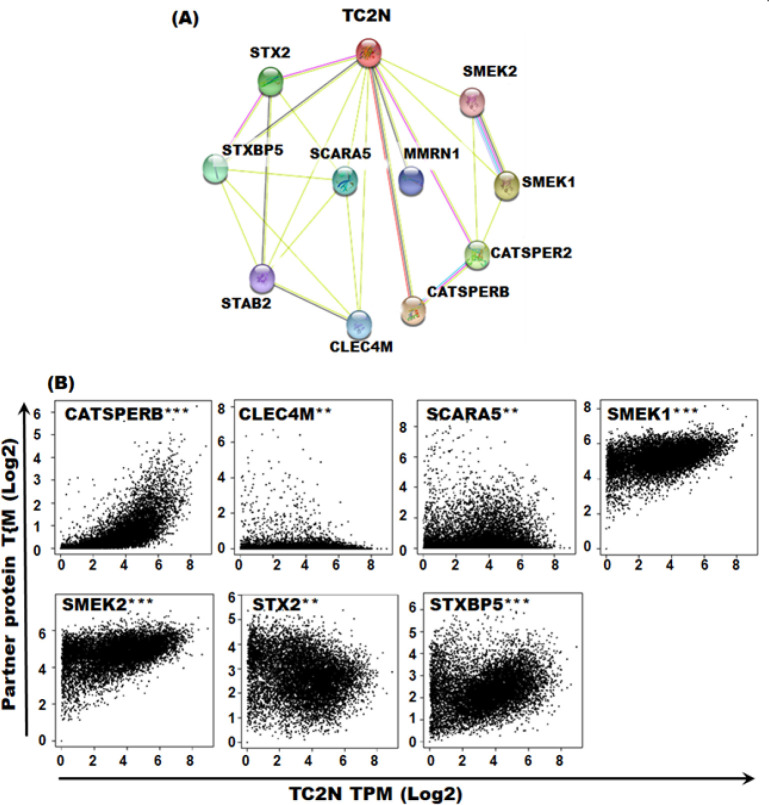
*TC2N* Protein Network. (A) TC2N functional protein partners were identified using STRING database by considering neighbourhood, gene fusion, co-expression, experiments and text mining approaches in the algorithm. Proteins with strongest interaction scores and direct interaction prediction are included in the figure. (B) Correlation between TC2N mRNA expression and mRNA expression levels of identified partner proteins was investigated in pan-cancer (cumulative correlation). ***p<0.0001, **p<0.01

In order to investigate potential functional partners of TC2N protein, we investigated STRING database. Proteins with strongest *TC2N* interaction scores included *STX2, SMEK2, SMEK1, STXBP5, SCARA5, MMRN1, CATSPER2, CATSPERB, CLEC4M* and *STAB2*. All these proteins were also identified in the list of genes showing +ve correlation with *TC2N* expression in at least one of the TCGA tumour types, further suggesting their potential role in carcinogenesis. Of the 10 proteins identified as functional TC2N partner, significant +ve correlation was observed between *TC2N* mRNA expression of *CATSPERB, CELEC4M, SCARA5, SMEK1,* *SMEK2, STX2 *and *STXBP*5 in pan-cancer (cumulative correlation), further suggesting that these proteins partner with TC2N in it’s signaling pathways (Figure 5). 

*TC2N mutation profile in pan-cancer*

**Figure 6:**
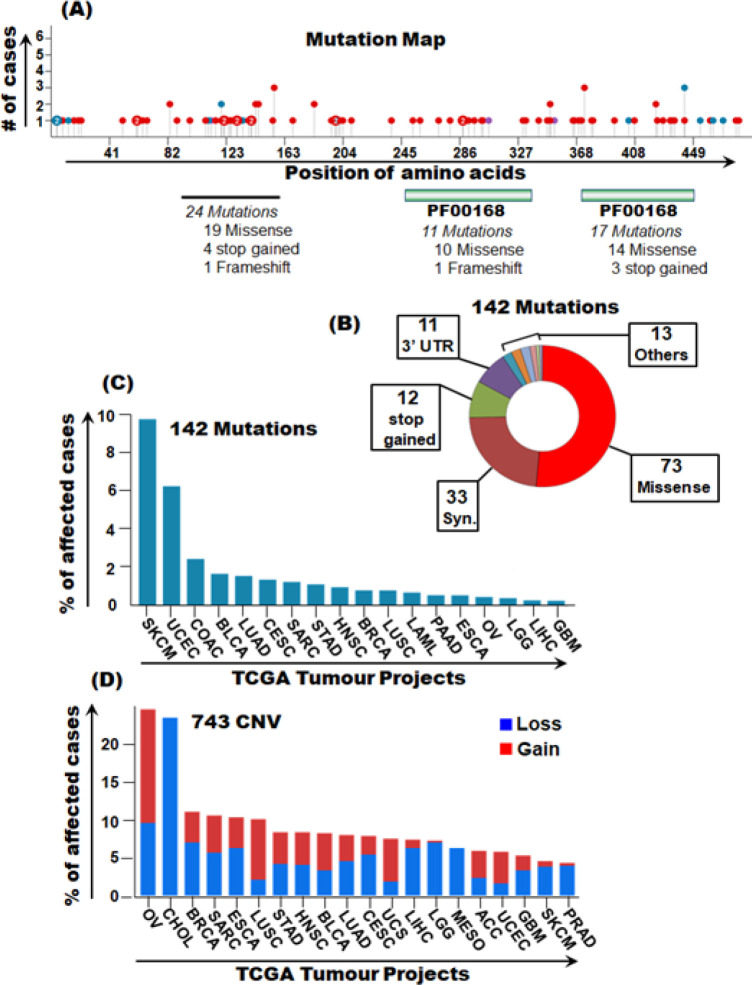
TC2N Somatic Mutations in Pan-Cancer. Mutation profiling of TC2N gene was undertaken using the GDC data portal. (A) Mutation map showing over all mutation burden of the TC2N gene in pan-cancer. (B) Pie chart describing types of 142 TC2N mutation in pan-cancer. (c) Percentage distribution of 142 TCN2 mutations in pan-cancer. (D) Percentage distribution of 743 CNVs in pan-cancer

Our analyses show several frequent somatic mutations in* TC2N* gene in various cancers. A total of 142 mutations were identified across 145 cases in a total of 18 TCGA tumour types. All 142 mutations were investigated for the type of mutation, and their potential of pathogenicity (FATHMM score>0.8). Of the 142 mutations, 73 were missense, 33 were synonymous, 12 were stop gained, 11 were in the 3’UTR and 13 were other types of mutations (Figure 6). Highest pathogenic mutation rates of TC2N were present in *SKCM, UCEC, COAD, BLCA* and *BRCA* (Table 3). In terms of copy number variants, burden of CNV gains was higher than CNV losses. Highest CNV gains were observed in *OV, BRCA, LUSC, HNSC, UCEC, BLCA, LUAD *and *STAD* while highest *CNV* losses were observed in *SARC, BLCA* and *ESCA* (Figure 6).

**Table 1:** TC2N Expression Patterns with Reference to Tumour Grade, Nodal Metastasis, Histological Subtype and Other Clinical Parameters

Tumour	Tumor grade	Nodal metastasis	Histological subtype	Other clinical
CHOL	N-vs-G: p=N/A N-vs-G2: p=0.06 N-vs-G3: p=0.07 N-vs-G4: p=1	N-vs-N0: p<10^-7^ N-vs-N1: p=0.03		
STAD	N-vs-G1: p=0.0008 N-vs-G2: p<10^-12^ N-vs-G3: p<10^-16^	N-vs-N0: p<10-^12^ N-vs-N1: p<10^-12^ N-vs-N2: p<10^-10^ N-vs-N3: p<10^-11^	N-vs-Adenocarcinoma (NOS): p<10-13 N-vs-Adenocarcinoma (Diffuse): p=<10-06 N-vs-Adenocarcinoma (Sigent Ring): p=0.007 N-vs-Intestinal Adenocarcinoma (NOS): p<10^-12^ N-vs-Intestinal Adenocarcinoma (Tubular): p<10^-11^ N-vs-Intestinal Adenocarcinoma (Mucinous): p=0.001 N-vs-Intestinal Adenocarcinoma (Papillary): p=0.01	T with H.pylori (↓ expression) -vs- T without H.pylori (↑ expression): p=0.0003
THCA		N-vs-N0: p<10^-11^ N-vs-N1: p<10^-12^	N-vs-Papillary carcinoma (classical): p<10^-10^ N-vs-Papillary carcinoma (tall): p<10^-12^ N-vs-Papillary carcinoma (follicular): p<10^-12^ N-vs-Others: p<10^-5^	
PCPG			N-vs-Paraganglioma: p=0.08 N-vs-Pheochromocytoma: p=0.4 N-vs-Paraganglioma;extra adrenal pheochromocytoma: p=0.004	

N, normal; p, p-value; G, grade; N/A, not applicable

**Table 2:** Expression Correlation between TC2N and Various Genes in Pan-Cancer

Genes with very strong +ve correlation with TC2N expression	DLBC: *BCL11B, CD28, CD3G, KIAA1671, TRAT1, GPRIN3, ITK, ABCA2, FYB, MAF, GIMAP7, GIMAP6, MBNL1, GIMAP2, PRKCQ, FYCO1, SLC46A3, ZNF75D , PLEKHM3, SH2D1A, INPP4B, OLFM2, GATA3, NTNG2, TNRC6C, FYN, NPHP3, GVIN1, PTPRC, ATM, EVI2B, STAT5B, ARL4C, ZNF831, MLL, SIRPG, LBH, WIPF1, SATB1.* KICH: *TMEM135, LIMA1, CDS1, OSBP, WDR20, PDP2, LONP2, KIAA0528, CLCN3, USP28, FCF1, TRIM2, LNX1, RALGAPA2, SETD3, ZYG11B, CDH1, SYNJ2BP, KIF16B.* LIHC: *STS, DSG2.* MESO: *LRRK2, ADRB2, RND1, SLC34A2, AGER, KIAA0040, FAM107A, AHCYL2, TACSTD2, SCGB3A1, AQP3, TMEM163, CCL20, SERPINA1, SOCS2, LAMP3, FCN3, LONRF3, HYAL1, GPIHBP1, FPR2, MPZL2, PRX, HOPX, CLDN7, CD101, PIGA, FMO5, LPL, CLDN4, SYTL3, CACNA2D2, EMP2, GK, ALPL, GLDN, CCL17, ABCA3, GPX3, AREG, SOX7, ICAM4, CYP4B1, FAM135A, LOC100302650, CH25H,EDN1, GRRP1, SFN, TACC2.* PCPG: *MUC1, TMEM30B, LLGL2, ERO1LB, MEIS2, SPINT1, NXT2, C2CD4C, SLC45A3, VAV1, ID4, ITPR3, CXADR, TMC4, C6orf192, ATP6V1B1, CHN2, DENND1C, PAX6, GLIS3, ZNF595, SLC25A15, GINS2, CNTD2, EPHB3, HOPX, MAGED4B, MEX3B, CDCA8, OTUB2, NCKAP1L.* PRAD: *CPSF2.* THYM: *BIRC3, FAM3B, CSF2RB, RAB11FIP4.* UCS: *GRHL2.*
Genes with strong –ve correlation with TC2N expression	CHOL: *ILVBL* DLBC: *RPS9* PRAD: *ZNF358* THYM: *ACD, FAM96B* UVM: *C19orf22, C19orf24, RAVER1*
+ve correlation b/w TC2N and various tumour suppressor genes in cancers	*BUB1B:* mod=PCPG; week=HNSC, READ, PRAD. *CYLD:* strong=DLBC, KICH; mod=PRAD, UCEC, READ, TGCT, UVM, LAML, THCA; week=LIHC, THYM, KIRC, KIRP, SKCM. *ATR*: strong=UVM, DLBC; mod=UCEV, KICH, LIHC, THCA, PAAD; week=BLCA, THYM, PRAD, KIRP. *ATM*: strong=DLBC; moderate=UVM CHK2; week=LAML, THYM, THCA, KIRP,PRAD,TGCT, HNSC, KIRC, LIHC, CESC,ESCA, *BRCA1*: week=CESC *BRCA2 *mod=CESC; week=HNSC, READ, DLBC, *TP53:* mod=HNSC.

**Table 3:** Pathogenic Somatic Mutations in *TC2N* Gene in Pan-Cancer

TCGA PROJECT	Genomic Position	Genetic Change	Type of Mutation	Protein Change	dbSNP ID/COSMIC ID
SKCM	91802368	G>A	Stop Gained	Q119*	rs766880081/COSM3498932
	91802395	G>A	Stop Gained	R110*	rs537836079,COSM2251654
	91785255	C>T	Misssense	M4231I	-/COSM5722247
	91800291	C>T	Missense	R184Q	rs753392127/COSM3498924
	91798325	C>T	Misssense	E238K	-/COSM3498924
	91812422	G>A	Misssense	S64F	-/COSM5540172
	91813715	C>T	Misssense	E19K	-/COSM4896986
	91799039	G>A	Misssense	P196L	-/COSM3498922
	91812464	G>A	Misssense	S50F	-/COSM4894496
	91785227	C>T	Misssense	E433K	-/COSM3498902
	91792368	G>A	Misssense	S349F	-/COSM958780
	91802311	G>A	Misssense	P138S	-/COSM3498930
	91797868	C>T	Misssense	D258N	-/COSM3498912
	91812435	C>T	Misssense	D60N	-/COSM3498936
	91799015	T>C	Misssense	N204S	-/COSM3498918
	91812324	C>T	Misssense	E97K	-/COSM3886531
	91802259	C>T	Misssense	G155E	-/COSM3498926
UCEC	91785197	G>A	Stop Gained	R443*	-/COSM958774
	91787558	G>T	Misssense	L373I	-/COSM958776
	91783191	C>T	Missense	S461N	-/COSM958772
	91802256	G>A	Missense	S156L	rs377688531/COSM198287
	91813760	C>A	Stop Gained	E4*	-/COSM958787
	91792368	G>A	Missense	S349F	-/COSM958780
	91812414	G>A	Missense	P67S	-/COSM958785
COAD	91785218	A>C	Missense	F436V	-/COSM277624
	91802256	G>A	Missense	S156L	rs37768531/COSM198287
	91785196	C>T	Missense	R433Q	rs117153533/COSM3690239
	91792369	A>G	Missense	S349P	-/COM270307
	91787582	T>G	Missense	N365H	-/COSM285615
BLCA	91787570	G>C	Missense	Q369E	-/COSM3793949
	91787540	G>T	Missense	P379T	-/COSM3793947
	91812366	A>G	Missense	S83P	-/COSM433482
BRCA	91802333	G>C	Missense	F130L	-/COSM1477845
	91799030	G>C	Missense	S199C	-/COSM3815523
	91812366	A>G	Missense	S83P	-/COSM433482
CESC	91792373	T>G	Missense	K347N	-/COSM4840762
LUAD	91792510	G>C	Missense	L302V	-/COSM3956286
STAD	91797805	A>C	Missense	S279A	-/COSM4052997
OV	91787543	G>A	Missense	L378F	-/COSM1323335
LGG	91802401	C>T	Missense	G108R	-/COSM3968969
LIHC	91798996	T>G	Missense	R210S	-/COSM4914987
GBM	91802256	G>A	Missense	S156L	-/COSM198287

## Discussion

Role of TC2N in cancers had been largely unaddressed until recently when it was identified as a novel oncogene in lung cancer where it works by inhibiting p53 signaling (Hao et al., 2019). Subsequently, the same group reported tumour suppressor role of* TC2N* in breast cancer (Hao et al., 2019). Nevertheless, role of TC2N in other caners remains unclear. In the study presented herein, we investigated molecular circuits of TC2N using the TCGA tumour data sets. 

We found that *TC2N* mRNA expression was upregulated in *CHOL, OV, READ, STAD* and* THYM*. It has been recently reported that increased *TC2N* transcript expression promotes cell proliferation and inhibits tumour cell apoptosis in lung cancer (Hao et al., 2019). It is therefore possible that TC2N perform similar pro-tumour functions in 5 tumour types identified herein with high TC2N mRNA levels. We also report increased mRNA expression in all stages, nodal metastasis and various histological types for CHOL and STAD suggesting a role of TC2N in the degree of malignancy for these tumours. It is therefore highly relevant to further investigate these findings using appropriately designed in-vivo and/or in-vitro assays. 

Levels of *TC2N* promoters were significantly higher in several cancers including the cancers for which we found downregulation of *TC2N* mRNA levels. Promoter hypermethylation is a key feature for transcriptional silencing of several genes in cancer (Park, 2010). In particular, tumour suppressor genes are silenced via hypermethylation in several caners (Nag and Yu, 2015). We also found *TC2N *promoter hypomethylation in HNSC and KIRC. There are evidence that hypomethylation may lead to increased genomic stability that may contribute towards carcinogenesis (Pfeifer, 2018). Moreover, DNA hypomethylation also leads to overexpression of proinvasive, antiapoptotic and angiogenic factors in prostate cancer (Vestergaar et al., 2010). In summary, TC2N promoter hyper and hypo-methylation are important findings of this study demanding further exploration. 

Genes which with similar functions and correlation almost always perform coordinated functions (Lee et al., 2014). In this study, we investigated correlation between *TC2N* mRNA expression and various cancer genes. Interestingly, many of the tumour types showed strong +ve and +ve correlation between *TC2N* genes and genes in common cancer pathways including Myc pathway, cell cycle pathway, *p53 *pathway, *Nrf2* pathway, Wnt pathway, Hippo pathway, *TGFβ *pathway, Notch pathway, *PI3K* pathway and *RTK/RAS* pathways. Some of the tumour types (such as *DLBC* and* PRAD*) showed +ve/strong +ve correlation of cancer causing genes with *TC2N* in almost all the cancer pathways investigated, strongly suggesting a potential role of *TC2N* in carcinogenesis of those tumours. 

Functional protein partners of *TC2N* were identified as *STX2, SMEK2, SMEK1, STXBP5, SCARA5, MMRN1, CATSPER2, CATSPERB, CLEC4M *and STAB2. Amongst these, there are some that warrants further investigation to delineate their partnership with *TC2N* in tumour microenvironment. For example, *CLEC4M* expression is downregulated in hepatocellular carcinoma and this downregulation is believed to promote inflammation and metastasis of HCC (Jovel et al., 2018). We also report downregulation of *TC2N* transcription in *PCPG, SKCM, THCA* and *UCS*. Another *TC2N* partner protein, *CATSPERB* has been linked with lung cancer. In a recent study that recruited 28 highly-aggregated-extended-highrisk-familial-lung-cancer (HRFLC) families, highest cluster of genetic variants associated with lung cancer were identified within *CATSPERB* gene (14q32) (Musolf et al., 2019). *SCARA5* plays an important role in tumourigenesis and metastasis of breast carcinoma by inhibiting *ERK1/2*, *STAT3* and *AKT* pathways (You et al., 2017). Taken together, several TC2N functional protein partners are involved in carcinogenesis, suggesting similar function (s) of *TC2N* in tumourigenesis. These findings therefore demand further functional studies in order to investigate/validate tumourigenic role of TC2N in various cancers. 

We identified a range of genetic alterations in the *TC2N* gene in several cancers. The highest pathogenic non-synonymous mutation rates were observed in *SKCM*, *UCEC, COAD, BLCA* and *BRCA*. Whether these genetic mutations are causative or a sequel of cancer processes needs to be investigated. It is important to note that cancer cells are susceptible to accumulate several mutations for multiple reasons such as increased cellular turnover, inflammatory tumour microenvironment, altered metabolic wiring, increased reactive oxygen species, increased susceptibility to DNA damage and decreased capacity of DNA damage repair amongst others (Loeb and Loeb, 2000; Hanahan and Weinberg, 2011; Fouad and Anani, 2017). Whether or not the mutations identified herein are passenger or driver mutations, needs to be explored further. 

Taken together, data presented in this study highlight several novel avenues with respect to *TC2N* role in carcinogenesis. Off course, more clinical and basic studies are required to further validate the findings presented herein. 

In conclusion, we present novel findings with respect to TC2N molecular circuitry in pan-cancer. Our analyses highlight potential role of *TC2N* in carcinogenesis of various cancers. Moreover, our findings also suggest TC2N interaction with many cancer genes in well-known cancer pathways. Taken together, we propose that TC2N may serve as an important player in carcinogenesis, prognosis and therapeutics of several cancers. It is therefore highly relevant to perform further clinical, *in-vitro *and/or *in-vivo *functional assays in order to validate these findings.

*List of Abbreviations*

ACC=adrenocortical carcinoma

BLCA=bladder urothelial carcinoma

BRCA=breast invasive carcinoma

CESC=cervical squamous cell carcinoma and endocervical adenocarcinoma

CHOL=cholangiocarcinoma

COAD=colon adenocarcinoma

DLBC=lymphoid neoplasm diffuse large B-cell lymphoma

ESCA=esophageal carcinoma

GBM=glioblastoma multiforme

HNSC=head and neck squamous cell carcinoma

KICH=kidney chromophobe

KIRC=kidney renal celar cell carcinoma

KIRP=kidney renal papillary cell carcinoma

LAML=acute myeloid leukemia

LGG=brain lower grade glioma

LIHC=liver hepatocellular

LUAD=lung adenocarcinoma

LUSC=lung squamous cell carcinoma

MESO=meshothelioma

OV=ovarian serious cystadenocarcinoma

PAAD=pancreatic adenocarcinoma

PCPG=pheochromocytoma and paraganlioma

PRAD=prostate adenocarcinoma

READ=rectum adenocarcinoma

SARC=sarcoma; SKCM=skin cutaneous melanoma

STAD=stomach adenocarcinoma

TGCT=testicular germ cell tumours

THCA=thyroid carcinoma

THYM=thymoma

UCEC=uterine corpus endometrial carcinoma

UCS=uterine carcinosarcoma

UVM=uveal melanoma

## Data Availability

The datasets analyzed during the current study are available at the “Genomic Data Commons Data Portal” (https://portal.gdc.cancer.gov/).
